# Predictors of severe COVID-19 among healthcare workers in Sabah, Malaysia

**DOI:** 10.1186/s12913-022-08920-4

**Published:** 2022-12-17

**Authors:** Lim Kai Joo, Mohd Fazeli Sazali, Michelle Goroh, Abraham Chin Zefong, Marilyn Charlene Montini Maluda, Richard Avoi, Valentine Japulee Gantul

**Affiliations:** 1 Sabah State Health Department, Ministry of Health Malaysia, Kota Kinabalu, Sabah Malaysia; 2 Penampang District Health Office, Sabah State Health Department, Ministry of Health, Kota Kinabalu, Malaysia; 3 Department of Public Health Medicine, Faculty of Medicine and Health Sciences, University Malaysia Sabah, Kota Kinabalu, Sabah Malaysia

**Keywords:** Severe COVID-19, Coronavirus Disease 2019, Healthcare worker, Predictors, Factors

## Abstract

**Background:**

Healthcare workers (HCWs) is the high-risk group for COVID-19 infection due to increased workplace exposure. However, evidence of the disease burden and factors associated with severe COVID-19 infection among HCWs is limited. Therefore, this article aims to describe the prevalence of severe COVID-19 disease among HCWs in Sabah, Malaysia, and to determine the factors associated with severe COVID-19 infection.

**Method:**

A retrospective cross-sectional study was carried out by assessing the data of COVID-19-infected HCWs in Sabah, Malaysia, from 1st March 2021 until 30th September 2021. Logistic regression analysis was used in this study.

**Results:**

Three thousand and forty HCWs were diagnosed with COVID-19 from 1st March 2021 until 30th September 2021. Of the 3040 HCWs, 2948 (97.0%) HCWs were mild, whereas 92 (3.0%) were severe. The multivariate logistic regression model showed that severe COVID-19 among HCWs in Sabah was associated with those do not receive any COVID-19 vaccination (aOR 6.061, 95% CI 3.408 – 10.780), underlying co-morbidity (aOR 3.335, 95% CI 2.183 – 5.096), and female (aOR 1.833, 95% CI 1.090 – 3.081).

**Conclusion:**

HCWs should strictly adhere to preventive measures, including vaccination, personal protective equipment, and early referral to a physician upon identifying severe COVID-19 infection. Early screening and aggressive co-morbidity treatment among HCWs are essential for public health practitioners to prevent severe COVID-19 disease. Regardless of co-morbidity status, HCWs should stay up to date with COVID-19 vaccination, including booster doses.

## Introduction

Since the pandemic of coronavirus disease 2019 (COVID-19) was declared by World Health Organization (WHO) on 11^th^ March 2020, the healthcare system has been severely impacted [1, 2]. All healthcare workers (HCWs) must work to face the pandemic, even during the national lockdown. These phenomena affect all HCWs, both physically and mentally. Various literature stated that the COVID-19 pandemic had been associated with psychological consequences for HCWs, including stress, depression, anxiety, trauma, burnout, and even suicidal attempts [1, 3–5]. Another study showed that 36.5% of the HCWs working in UMMC experienced anxiety symptoms, while 29.5% reported depressive symptoms [4]. These harmful consequences of the pandemic on workers' mental health have prompted initiatives to prevent and control the problem through various mental health programs and religious coping [4, 5].

Besides being psychologically impacted, HCWs are also often considered a high-risk population for acquiring the infection as this population has frequent contact with COVID-19 patients and the risky working environment, such as treating patients in the indoor setting [6]. Despite becoming more resilient and improving occupational intervention to prevent infection among HCWs [7, 8], illness and death have profoundly affected the HCW. The previous report from the Italian health authority stated that 10.7% of overall COVID-19 cases were contributed by HCWs, with a rising number of deaths among medical doctors, nurses, nurse aides, and dentists [7]. In a large register-based cohort study among Scottish healthcare workers, HCWs and their households contributed to a sixth of COVID-19 cases [8]. A cohort study in the United Kingdom (UK) found that the essential workers, including HCWs, the risk of acquiring severe COVID-19 infection seven times greater compared to the non-essential workers (RR 7.43, 95% CI 5.52 to 10.00) [9]. Furthermore, the risk of disease among HCWs was more remarkable due to unprotected exposure to the infected individuals, involvement in aerosol-producing procedures, and contact with bodily secretions [10].

Severe COVID-19 infection can lead to long-term complications, including multisystem inflammatory syndrome, pulmonary fibrosis, and neurological manifestations [11–16]. Even though there is greater awareness and strict adherence to infection control measures, HCWs had a higher risk of severe COVID-19 infection than other occupations and general populations [17, 18]. Hence, it is crucial to study the factors associated with severe COVID-19 disease in HCWs. Therefore, this article aims to describe the prevalence of severe COVID-19 infection among HCWs in Sabah, Malaysia, and to determine the factors associated with severe COVID-19 disease.

## Methods

### Study design, location, and duration

A retrospective cross-sectional study was carried out by assessing the data of COVID-19-infected healthcare workers (HCWs) in Sabah, Malaysia from 1^st^ March 2021 until 30^th^ September 2021. Sabah is one of the biggest states in Malaysia, situated in the northern portion of Borneo Island. It shares its border with the Malaysian state of Sarawak, Vietnam, the Philippines, and Indonesia. Sabah has a geographical area of 73,904 km2 and is divided into 25 districts. The state's population is 3.9 million and it is among the state with lowest population density in the country (44 people/km2).

### Study population

The study population involved all laboratory confirmed-COVID-19 infection among HCWs working in public healthcare facilities in Sabah that was registered from 1st March 2021 until 30th September 2021. The study was conducted when the COVID-19 vaccine (Pfizer BioNTech©) was available in Malaysia in February 2021. Thus, secondary data of infected HCWs after vaccination was available was collected. Registered infected HCWs represent all occupational categories working in Sabah government health facilities and confirmed by laboratory tests. Confirmed COVID-19 infection among HCWs was reported by the Occupational Safety and Health (OSH) unit from various government health facilities across Sabah state, including 24 hospitals, 25 area health offices, nine area dental offices, eight headquarters, and one public health laboratory in Kota Kinabalu, Sabah.

### Data collection instruments and procedure

The data of HCWs that have been registered and confirmed COVID-19 were obtained from Public Health Division, Sabah State Health Department. The data contain sociodemographic characteristics, clinical characteristics, co-morbidity, occupational categories, COVID-19 patient work involvement, and probable source of infection. The data was collected through an investigation conducted by trained district health officials through phone call interviews. Two public health professionals provided continuous supervision and monitoring to ensure the quality of information collected to ensure completeness and consistency of data collected.

Data in patients' line-listing were extracted in Microsoft Excel format from 1st March 2021 until 30th September 2021. Data extracted were cleaned to remove any irrelevant observations before analyses were performed. All registered and confirmed COVID-19 HCWs in government health facilities were included, and any probable cases or notified but not registered cases were excluded from the analysis. The data analysis was done using Statistical Package for Social Science (SPSS version 22.0) software. The descriptive data were presented as mean ± standard deviation (SD). Univariate and multivariate logistic regression were used to analyze factors associated with COVID-19 severity. A value of *p* < 0.05 is considered statistically significant.

### Outcome measures and operational definitions

Confirmed COVID-19 infections can be defined as a person who is either alive or dead with positive real-time reverse transcriptase polymerase chain reaction (RT-PCR) or a person who has positive rapid test kit antigen (RTK-Ag) in pre-determined areas/locality with the prevalence of COVID-19 > 10% [19]. The clinical specimens sent for RT-PCR and RTK-Ag were obtained from the nasopharyngeal swabs, sputum, tracheal aspiration, or bronchoalveolar lavage.

For vaccination status, complete vaccination means those HCWs who have completed two vaccination doses. The protected period was valid if the disease onset was after 14 days from the second dose vaccination. Whereas incomplete vaccination means the recipient only received one dose of vaccine or two doses but less than the reasonable protective period, 14 days.

Under the staff categories, there are several worker positions such as nurses, doctors, PPP (*Penolong Pegawai Perubatan* or Assistant Medical Officer), PPK (*Pembantu Perawatan Kesihatan* or Health Assistant), PPKP (*Penolong Pegawai Kesihatan Persekitaran* or Assistant Environmental Health Officer), PKA (*Pembantu Kesihatan Awam* or Public Health Assistant), Pharmacy staffs, dental staffs, and other workers categories. The PPP has a vital role as a paramedic to provide primary health care, accident and emergency services, administrative, and specific roles under certain departments such as orthopaedics, surgery, psychiatry, etc. Meanwhile, PPK's general function is as a basic healthcare provider, such as ensuring patients comfort, making patients' beds, preparing medical equipment, etc. PPKP and PKA have a public health role in investigating environmental or health hazards and subsequently taking action to eliminate or limit the risk.

For the 'probable source of infection' variable, the question was asked to the patient on how they got the infection, either from their patient or other healthcare staff (work related); or from their family members, housemates, or through social interaction (non-work related). Meanwhile, For the 'COVID-19 involvement' variable, the patients were asked if they had to manage any COVID-19 patients during the incubation period. Co-morbidities variable refers to any reported underlying health problems that their doctor diagnosed. The health problems include but are not limited to hypertension, diabetes, pregnancy, obesity, chronic lung disease, asthma, malignancy, HIV, chronic kidney disease, and chronic liver disease.

The study used mild and severe COVID-19 infection as binary outcome measures. Mild COVID-19 infection represents stages 1 and 2, whereas severe cases represent stages 3,4 and 5. The staging of COVID-19 infection was made through clinical evaluation by a clinical specialist combined with laboratory and imaging studies assessment. Each case was staged according to clinical severity; stage I: asymptomatic, stage II: symptomatic without pneumonia, stage III: pneumonia without hypoxia, stage IV: pneumonia with hypoxia requiring oxygen supplementation therapy and stage V: critically ill. The diagnosis and staging made were according to COVID-19 Management Guidelines in Malaysia [19].

## Results

Three thousand forty (3040) healthcare workers (HCWs) were diagnosed with COVID-19 from 1^st^ March 2021 until 30^th^ September 2021. Of the 3040 HCWs, 2948 (97.0%) HCWs were mild, whereas 92 (3.0%) were severe (Table 1). About 2821 (92.8%) of the HCWs were vaccinated completely compared to incomplete vaccination (92, 3.0%) and not vaccinated at all (127, 4.2%), even though they were eligible. Among the positive HCWs, most were female (68.5%). HCWs with no co-morbidity contributed 2234 (73.5%) compared to those with co-morbidity, 806 (26.5%). Among the positive HCWs, nurses contributed the highest number, which is 1271 (41.8%), followed by medical assistants 527 (17.3%), doctors (256, 8.4%), inspectorate (146, 4.8%), dental (122, 4.0%), pharmacy (87, 2.9%) and other categories 631 (20.8%) such as administration, engineers, and laboratory personnel. Non-work related positive HCWs contributed 2383 (78.4%), and work-related were 657 (21.6%).

**Table 1:** General Characteristics of Confirmed COVID-19 Healthcare Workers in Sabah

Variables	Frequency (*n* = 3040)	Percentage (100%)
Staging		
Mild	2948	97.0%
Severe	92	3.0%
Age	Mean 35.91, S.D 7.869, min 19, max 60	
Age Group		
40 years and below	2302	75.7%
Above 40 years old	738	24.3%
Gender		
Female	2083	68.5%
Male	957	31.5%
COVID-19 Vaccination		
Not at all	127	4.2%
Incomplete vaccination	92	3.0%
Complete vaccination	2821	92.8%
Co-morbid		
No	2234	73.5%
Yes	806	26.5%
Staff Categories		
Nurses	1271	41.8%
Doctors	256	8.4%
PPP/PPK^a^	527	17.3%
PPKP/PKA^a^	146	4.8%
Pharmacy	87	2.9%
Dental	122	4.0%
Others	631	20.8%
Probably source of infection		
Non-work related	2383	78.4%
Work-related	657	21.6%
COVID-19 Work Involvement		
No	1808	59.5%
Yes	1232	40.5%

^a^PPP *Penolong Pegawai Perubatan* (Assistant Medical Officer), *PPK Pembantu Perawatan Kesihatan* (Health Assistant), *PPKP Penolong Pegawai Kesihatan Persekitaran* (Assistant Environmental Health Officer), *PKA Pembantu Kesihatan Awam* (Public Health Assistant)

The dependent variable in this study is the staging of COVID-19 disease, which is mild and severe. There are seven independent variables: age, gender, staff categories, COVID-19 vaccination status, co-morbid, involvement in COVID-19 work, and source of infection. From the univariate logistic regression (Table 2), four variables are considered included in the multivariable logistic regression model using *p*-value < 0.25: age, gender, COVID-19 vaccination status, and co-morbidity.

**Table 2:** Univariable Logistic Regression Analysis

Predictors	COVID-19 cases, N (row %)		Total		
	Mild	Severe		Unadjusted OR (95% CI)	*p*-value
COVID-19 Staging	2948 (97.0)	92 (3.0)	3040	-	-
Age*			3040	1.029 (1.004 – 1.055)	0.022
Gender*					
Male	938 (98.0)	19 (2.0)	957	1	
Female	2010 (96.5)	73 (3.5)	2083	1.793 (1.076 – 2.988)	0.025
Staff Categories					
Nurses	1220 (96.0)	51 (4.0)	1271	1	
Doctors	249 (97.3)	7 (2.7)	256	0.672 (0.302 – 1.499)	0.332
PPP/PPK^a^	511 (97.0)	16 (3.0)	527	0.749 (0.423 – 1.326)	0.321
PPKP/PKA^a^	143 (97.9)	3 (2.1)	146	0.502 (0.155 – 1.629)	0.251
Pharmacy	84 (96.6)	3 (3.4)	87	0.854 (0.261 – 2.795)	0.795
Dental	121 (99.2)	1 (0.8)	122	0.198 (0.027 – 1.443)	0.110
Others	620 (98.3)	11 (1.7)	631	0.424 (0.220 – 0.820)	0.011
COVID-19 vaccination*					
Complete	2751 (97.5)	70 (2.5)	2821	1	
Incomplete	87 (94.6)	5 (5.4)	92	2.259 (0.889 – 5.736)	0.087
Not at all	110 (86.6)	17 (13.4)	127	6.074 (3.458 – 10.667)	0.000
Co-morbid*					
No	2190 (98.0)	44 (2.0)	2234	1	
Yes	758 (94.0)	48 (6.0)	806	3.152 (2.076 – 4.784)	0.000
COVID-19 Involvement					
No	1750 (96.8)	58 (3.2)	1808	1	
Yes	1198 (97.2)	34 (2.8)	1232	0.856 (0.557 – 1.316)	0.479
Probably Source					
Non-work related	2311 (97.0)	72 (3.0)	2383	1	
Work-related	637 (97.0)	20 (3.0)	657	1.008 (0.609 – 1.667)	0.976

^*^p-value ≤ 0.25 variables included into multivariate logistic regression

^a^PPP *Penolong Pegawai Perubatan* (Assistant Medical Officer), *PPK Pembantu Perawatan Kesihatan* (Health Assistant), *PPKP Penolong Pegawai Kesihatan Persekitaran* (Assistant Environmental Health Officer), *PKA Pembantu Kesihatan Awam* (Public Health Assistant)

After the four variables (age, gender, COVID-19 vaccination, and co-morbid) were included in the multivariable logistic regression model, only three variables were statistically significant (*p*-value ≤ 0.05), which is gender, COVID-19 vaccination and co-morbid (Table 3). The multivariable logistic regression model showed that severe COVID-19 among HCWs in Sabah was associated with those do not receive any COVID-19 vaccination (aOR 6.061, 95% CI 3.408 – 10.780), underlying co-morbidity (aOR 3.335, 95% CI 2.183 – 5.096), and female (aOR 1.833, 95% CI 1.090 – 3.081. The model has a satisfactory fit with a classification table of 97%.

**Table 3:** Multivariable Logistic Regression Model

Predictors	Multivariate Logistic Regression	
	Adjusted OR (aOR) (95% CI)	*p*-value
Gender*		
Male	1	
Female	1.833 (1.090 – 3.081)	0.022
COVID-19 Vaccination		
Complete	1	
Incomplete	2.254 (0.876 – 5.803)	0.092
Not at all*	6.061 (3.408 – 10.780)	0.000
Co-morbid*		
No	1	
Yes	3.335 (2.183 – 5.096)	0.000
Age	1.014 (0.988 – 1.040)	0.289

^*^p-value ≤ 0.05

## Discussion

Severe COVID-19 infection among healthcare workers (HCWs) in Sabah contributed to 92 cases from 3040 total COVID-19 diseases in this study which is 3.0%. The incidence of severe COVID-19 illness among the current study population is lower than in previous studies, with around 5% to 9.9% incidence of severe disease among HCWs, as reported in meta-analyses [20, 21]. The occurrence of severe infection in the current study is also lower than the reported severe infection among all COVID-19-positive patients. For example, a survey among hospitalized patients in France reported that 41% with severe diseases, including 32% requiring ICU admission and 11% deaths [22]. A similar finding also was documented in a study among patients admitted to a tertiary medical centre, where 74 (37.6%) patients required intubation [23]. The inconsistency of the prevalence is because HCWs might have better awareness regarding COVID-19 infection. Furthermore, greater access to testing facilities also enables the population to be identified earlier, allowing early treatment of the disease.

The current study identified several independent risk factors associated with severe COVID-19 infection among HCWs. The female gender is significantly associated with a greater risk of having severe COVID-19 disease than the male. However, the finding is inconsistent with previous studies that identified infected males as more likely to get severe COVID-19 infection than females [22, 24–27]. Conflicting results in the current study might be explained by the imbalance in proportions of sex differences among all COVID-19 patients and among HCW. In the present study population, almost 70% of infected HCWs were female, most of whom were nursing staff. The more significant proportion of nursing staff in the current study might hinder the effect of the male gender on severe COVID-19 infection. Furthermore, the previous study also found the association of the male gender with severe COVID-19 illness is weak [25–28]. In a physiological context, females have a higher copy number of immune-related genes, providing a more robust immune response, leading to high-stress endurance levels and better viral clearance. Consequently, lower mortality was observed [29].

Co-morbidity was also significantly associated with severe COVID-19 infection among HCWs. The current study found that HCWs with co-morbidity have a three times greater risk of acquiring severe COVID-19 infection than those without co-morbidity. Previous studies reported that co-morbidity such as immunosuppression [22, 30], diabetes [22, 25, 27, 28, 30, 31], hypertension [26, 28], acute or chronic kidney disease [22, 23], chronic obstructive pulmonary disease [31], bronchial asthma [25, 30], and gastrointestinal condition [27, 28, 30] were associated with severe COVID-19 infection and death. Furthermore, the previous study also similarly found that patients with haematological co-morbidities such as acute myeloid leukaemia (3·49, 1·56–7·81), indolent non-Hodgkin lymphoma (2·19, 1·07–4·48), aggressive non-Hodgkin lymphoma (2·56, 1·34–4·89), and plasma cell neoplasms were associated with worse COVID-19 survival [32]. Previous meta-analyses found that severity is most remarkable in patients with a history of cerebrovascular disease: OR 4.85 (95% CI: 3.11–7.57), cardiovascular disease: OR 4.81 (95% CI: 3.43–6.74), chronic lung disease: OR 4.19 (95% CI: 2.84–6.19), and cancer: OR 3.18, 95% CI: 2.09–4.82 [33]. The high prevalence of co-morbidity among infected HCWs in our study requires urgent intervention to protect the affected HCWs from serious sequelae of COVID-19 infection, including long COVID-19 syndrome, acute respiratory distress syndrome (ARDS), multi-organ failure, septic shock, and death [34, 35].

Vaccination is one of the essential public health interventions to prevent severe COVID-19 infection. The current study found that HCWs who are unvaccinated or had incomplete vaccination history are more likely to have severe COVID-19 disease by two to six times respectively, than those who had completed COVID-19 vaccination. The finding is supported by a previous study that found that receiving at least two doses of vaccination reduces the risk of hospital admission by 93%, 92% for severe COVID-19, and 81% for COVID-19-related deaths [36]. This finding further underscores the importance of vaccination among HCWs and those with co-morbidities [37]. A recent study also highlights the importance of third-dose mRNA vaccination in protecting individuals against severe COVID-19-related outcomes [36].

Previous studies among the general population have found that advancing age, especially greater than 60 years old, was an independent risk factor for severe COVID-19 infection and deaths [22, 25–27]. The elderly population is at high risk for severe COVID-19 disease because they have weakened immunity and viral-induced cytokine storms, resulting in life-threatening respiratory failure and multisystemic involvement [38]. Since HCWs are among productive age groups, the age factor was not significantly associated with severe COVID-19 infection in the current study. In the present study, even though the age factor is not a significant concern, these populations must be regularly screened for co-morbidity, especially before retirement, to protect them when they enter high-risk age groups. The current study also found that the various HCW professions and probable sources of infection were not significantly associated with severe COVID-19 disease. However, in past literature, it was observed that HCWs working at the front door, intensive care unit and aerosol-generating procedure settings were associated with hospital admission [8]. The inconsistency of findings with the current study might be because the present study did not stratify the staff's background, as some HCWs might not work directly with COVID-19 patients. Furthermore, the current study also identified that most of the infection originated from non-work-related conditions.

From the study, there are several recommendations. Assessing awareness and practice regarding preventive measures against COVID-19 might help determine the need for tailored health education programs among HCWs to improve the appropriate method of COVID-19 prevention practices [39]. With the advancement of technology and computer science, the current surveillance system for early detection of COVID-19 infection among HCWs could be improved through disruptive technology such as the Internet of things, artificial intelligence, and blockchain [40]. Previous research has reported that ventilation is essential in transmitting infectious diseases, such as severe acute respiratory syndrome and influenza [41]. Therefore, a future study in assessing indoor air quality in healthcare settings might be needed to determine the potential neglected source of infection with subsequent intervention to improve ventilation. For HCWs, strict adherence to preventive measures, including vaccination, early and self-referral to the physician upon identification of severe COVID-19 infection may help to prevent further progression and complications of COVID-19. For public health practitioners, early screening and treatment of co-morbidity are of utmost importance for preventing severe COVID-19 infection among HCWs. HCWs with uncontrolled co-morbidities are the group to be prioritized to get the vaccination. Occupational health policy emphasizing the protection of HCWs from COVID-19 infection should be prioritized in public and private healthcare facilities.

This study is not without limitations. A cross-sectional study design where the data was collected at a single time point and the association between predictors and outcome may not be taken as an indication of causality, and reverse causality is possible. Furthermore, because of the retrospective nature of this study, some of the critical variables, especially potential confounders such as the laboratory, inflammatory, infection markers, and behavioural risk factors, were unavailable. In addition, a possible lack of detailed clinical data and self-reporting of co-morbidity could limit our findings. Thus, some variables' role in predicting severe COVID-19 infection could have been underestimated. Nonetheless, the heterogenicity of our study population, involving all healthcare staff regardless of whether they work in clinical or non-clinical practice, provides valuable information on potential predictors of severe disease in the population.

## Conclusion

In conclusion, the study has elucidated the list of vulnerable populations among HCWs that predicted severe COVID-9 infection, including gender, vaccination status, and co-morbidity. The findings from our study have several implications for various stakeholders, such as HCWs, public health practitioners, policymakers, physicians at healthcare facilities, and intensive care physicians. Regardless of the co-morbidity status, the HCW are recommended to stay up to date with their COVID-19 vaccination, including booster doses. Strict adherence to preventive measures and occupational health policy is of the utmost importance to prevent severe COVID-19 infection among HCWs.

## Data Availability

The datasets obtained for this study are not publicly available to protect patients' confidentiality. Any interested parties may request related information from Malaysian Ministry of Health or directly from the first author (carolimkj@gmail.com) on reasonable request.
